# Probabilistic and Systematic Coverage of Consecutive Test-Method Pairs for Detecting Order-Dependent Flaky Tests

**DOI:** 10.1007/978-3-030-72016-2_15

**Published:** 2021-03-01

**Authors:** Anjiang Wei, Pu Yi, Tao Xie, Darko Marinov, Wing Lam

**Affiliations:** 8 Eindhoven University of Technology, Eindhoven, The Netherlands; 9 Aalborg University, Aalborg East, Denmark; 10 Peking University, Beijing, China; 11 University of Illinois at Urbana-Champaign, Urbana, IL USA

**Keywords:** Flaky tests, Order dependent, Test-pair coverage

## Abstract

Software developers frequently check their code changes by running a *set* of tests against their code. Tests that can nondeterministically pass or fail when run on the same code version are called *flaky tests*. These tests are a major problem because they can mislead developers to debug their recent code changes when the failures are unrelated to these changes. One prominent category of flaky tests is order-dependent (OD) tests, which can deterministically pass or fail depending on the *order* in which the set of tests are run. By detecting OD tests in advance, developers can fix these tests before they change their code. Due to the high cost required to explore all possible orders (*n*! permutations for *n* tests), prior work has developed tools that randomize orders to detect OD tests. Experiments have shown that randomization can detect many OD tests, and that most OD tests depend on just one other test to fail. However, there was no analysis of the probability that randomized orders detect OD tests. In this paper, we present the first such analysis and also present a simple change for sampling random test orders to increase the probability. We finally present a novel algorithm to systematically explore all consecutive pairs of tests, guaranteeing to detect all OD tests that depend on one other test, while running substantially fewer orders and tests than simply running all test pairs.

### 1 Introduction

The most common way that developers check their software is through frequent regression testing performed while they develop software. Developers run regression tests to check that recent code changes do not break existing functionality. A major problem for regression testing is *flaky tests* [27], which can nondeterministically pass or fail when run on the same code version. The failures from

** Tao Xie is with the Key Laboratory of High Confidence Software Technologies (Peking University), Ministry of Education, China, and is the corresponding author.

these tests can mislead developers to debug their recent changes while the failures can be due to a variety of reasons unrelated to the changes. Many software organizations have reported flaky tests as one of their biggest problems in software development, including Apple [18], Facebook [5,10], Google [8,30,31,43,48], Huawei [16], Microsoft [11,12,20,21], and Mozilla [40].

These flaky tests are among the tests, called *test suite*, that developers run during regression testing; a test suite is most often specified as a *set*, not a sequence, of tests. Having a test suite as a set provides benefits for regression testing techniques such as selection, prioritization, and parallelization [23,45]. The test execution platform can choose to run these tests in various *test orders*. For example, for projects using Java, the most popular testing framework is JUnit [17], and the most popular build system is Maven [28]. Tests in JUnit are organized in a set of *test classes*, each of which has a set of *test methods*. By default, Maven runs tests using the Surefire plugin [29], which does not guarantee any order of test classes or test methods. However, the use of Surefire and JUnit does *not* interleave the test methods from different test classes in a test order. The same structure is common for many other testing frameworks such as TestNG [41], Cucumber [4], and Spock [38].

One prominent category of flaky tests is deterministic *order-dependent (OD) tests* [22,24,32,47], which can deterministically pass or fail in various test orders, with at least one order in which these tests pass and at least one other order in which they fail. Other flaky tests are non-deterministic (ND) tests, which are flaky due to reasons other than solely the test order [24]; for at least one test order, these tests can nondeterministically pass or fail even in that same test order. Our iDFlakies work [22] has released the iDFlakies dataset [15] of flaky tests in open-source Java projects. We obtained this dataset by running test suites many times in randomized test orders, collecting test failures, and classifying failed tests as OD or ND flaky tests. In total, 50.5% of the dataset are OD tests, while the remaining 49.5% are ND tests.

Prior research has proposed multiple tools [2,6,9,14,22,47] to detect OD tests. Some of the tools [9,14] search for *potential* OD tests and may therefore report false alarms, i.e., tests that cannot fail in the current test suite (but may fail in some extended test suite). The other tools [2,6,22,47] detect OD tests that actually fail by running multiple randomized orders of the test suite. Running tests in random orders is also available in many testing platforms, e.g., Surefire for Java has a mode to randomize the order of test classes, `pytest` [35] for Python has the `--random-order` option, and `rspec` [36] for Ruby has the `--order random` option. While these tools can detect many OD tests, the tools run random orders and hence can miss running test orders in which OD tests would fail. The listed prior work has *not* studied the *flake rates*, i.e., the probability that an OD test would fail when run in (uniformly) sampled test orders.

Our iFixFlakies work [37] has studied the *causes* of failures for OD tests. We find that the vast majority of OD tests are related to *pairs* of tests, i.e., each OD test would pass or fail due to the sharing of some global state with just one other test. Our iFixFlakies work has also defined multiple kinds of tests related to OD tests. Each OD test belongs to one of two kinds: (1) *brittle*, which is a test that fails when run by itself but passes in a test order where the test is preceded by a *state-setter*; and (2) *victim*, which is a test that passes when run by itself but fails in a test order where the test is preceded by a (state-)*polluter* unless a (state-)*cleaner* runs in between the polluter and the victim. Most of the work in this paper focuses on victim tests because most OD tests are victims rather than brittles (e.g., 91% of the truly OD tests in the iDFlakies dataset are victims [15]), and the analysis for brittles often follows as a simple special case of the analysis for victims.

This paper makes the following two main contributions.

**Probability Analysis.** We develop a methodology to analytically obtain the flake rates of OD tests and propose a simple change to the random sampling of test orders to increase the probability of detecting OD tests. A flake rate is defined as the ratio of the number of test orders in which an OD test fails divided by the total number of orders. Flake rates can help researchers analytically compare various algorithms (e.g., comparing reversing a passing order to sampling a random order as shown in Section 4.4) and help practitioners prioritize the fixing of flaky tests. Specifically, we study the following problem: determine the flake rate for a given victim test with its set of polluters and a set of cleaners for each polluter. We first derive simple formulas with two main assumptions: (A1) all polluters have the same set of cleaners and (A2) all of the victim, polluters, and cleaners are in the same test class. We then derive formulas that keep A1 but relax A2. Our results on 249 real flaky tests show that our formulas are applicable to 236 tests (i.e., only 13 tests violate A1). To relax both assumptions, we propose an approach to estimate the flake rate without running test orders. Our analysis finds that some OD tests have a rather low flake rate, as low as 1.2%.

**Systematic Test-Pair Exploration.** Because random sampling of test orders may miss test orders in which OD tests fail, we propose a systematic approach to cover all consecutive test pairs to detect OD tests. We present an algorithm that systematically explores all consecutive test pairs, guaranteeing the detection of all OD tests that depend on one other test, while running substantially fewer tests than a naive exploration that runs every pair by itself. Our algorithm builds on the concept of Tuscan squares [7], studied in the field of combinatorics. Given a test suite, the algorithm generates a set of test orders, each consisting of at least two distinct tests and at most all of the tests from the test suite, that cover all of the consecutive test pairs, while trying to minimize the cost of running those test orders. The algorithm can cover pairs of tests from the same and different classes, while considering only the test orders that do not interleave tests from different test classes, being a common constraint of testing frameworks such as JUnit [17]. Our analysis shows that the algorithm runs substantially fewer tests than naive exploration. To experiment with the new algorithm based on Tuscan squares, we run some of the test orders generated by the algorithm for some of the test suites in the iDFlakies dataset. Our experiments detect 44 new OD tests, not detected in prior work [22,24,25], and we have added the newly detected tests to the Illinois Dataset of Flaky Tests [19].

```java
public void testMRAppMasterSuccessLock() { // testV for short
  ... // setup MapReduce job, e.g., set conf and userName
  MRAppMaster appMaster =
      new MRAppMasterTest("appattempt_...", "container_...", "host", -1,
        -1, System.currentTimeMillis(), false, false);
  try {
    MRAppMaster.initAndStartAppMaster(appMaster, conf, userName);
  } catch (IOException e) { ... }
  ... // assert the state and some properties of appMaster
  appMaster.stop();
}
```

**Fig. 1.** Victim OD test from Hadoop's `TestMRAppMaster` class.

```java
public void testSigTermedFunctionality() { // testP for short
  JHEventHandlerForSigtermTest jheh =
    new JHEventHandlerForSigtermTest(Mockito.mock(AppContext.class), 0);
  jheh.addToFileMap(Mockito.mock(JobId.class));
  ... // have jheh handle a few events
  jheh.stop();
  ... // assert whether the events were handled properly
}
```

**Fig. 2.** Polluter test from Hadoop's `TestJobHistoryEventHandler` class.

### 2 Background and Example

We use an example to introduce some key concepts for OD tests and to illustrate challenges in debugging these tests. We represent a test order as a sequence of tests $\langle t_1, t_2, \ldots, t_l \rangle$. In Java, each test order is executed by a Java Virtual Machine (JVM) that starts from the initial state (e.g., all shared pointer variables initialized to `null`) and then runs each test, which potentially modifies the shared state. Each test is run *at most once* in one JVM run. (Thus, covering test orders and test pairs has to be done with a *set* of test orders and cannot be done with just one very long order, e.g., using superpermutations [13].) A test $v$ is a *victim* if it passes in the order $\langle v \rangle$ but fails in another order; the other order usually contains a single *polluter* test $p$ (besides many other tests) such that $v$ fails even in the order $\langle p, v \rangle$. Moreover, the test suite may contain a *cleaner* test $c$ such that $v$ passes in the order $\langle p, c, v \rangle$. Note that test orders *may contain more tests* besides polluters and cleaners for a victim $v$, but these other tests do not modify the relevant state and do not affect whether $v$ passes or not in any order. Precise definitions for these tests are in our previous work [37].

Figure 1 shows a snippet of a victim test, `testMRAppMasterSuccessLock` (in short `testV`), from the widely used Hadoop project [1]. The test suite for this test has 392 tests. This test is from the MapReduce (MR) framework and aims to check an MR application. This test is a victim because it passes when run by itself but has two polluter tests. If the victim is run after either one of its polluter tests (and no cleaner runs in between the polluter and the victim), then the victim fails with a `NullPointerException`. Figure 2 shows a snippet of one of these two polluter tests, `testSigTermedFunctionality` (in short `testP`).

These tests form a polluter-victim pair because they share a global state, namely all "active" jobs stored in a *static* map in the `JobHistoryEventHandler`

class. (In JUnit 4, only the heap state reachable from the class fields declared as static is shared across tests; JUnit does *not* automatically reset that state, but developers can add `setup` and `teardown` methods to reset the state.) To check an MR application, `testV` first sets up some state (Line 2), then creates an MR application (Line 3), and starts the application (Line 7). The `NullPointerException` arises when the test tries to stop the MR application (Line 10). Specifically, the `appMaster` accesses the shared map data structure that tracks all jobs run by any application. When `testV` is run after `testP`, then `appMaster` will attempt to stop a job created by the polluter, although the job has already been stopped.

This static map is empty when the JVM starts running a test order, and it is also explicitly cleared by some tests. In fact, we find 11 cleaner tests that clear the map, and the victim passes when any one of these 11 tests is run between `testP` and `testV`. Interestingly, for the other polluter test, `testTimelineEventHandling` (in short `testP′`), the victim fails for the same reason, but `testP′` has 31 cleaners—the same 11 as `testP` and 20 other cleaners. Our manual inspection finds that the `testP′` polluter has other cleaners because the job created by `testP′` is named `job_200_0001`, while the job created by the `testP` polluter is a mock object. The 20 other cleaners also create and stop jobs named `job_200_0001` and therefore act as cleaners for the `testP′` polluter but not the `testP` polluter. This example illustrates not only how victims and polluters work but also the complexity in how these tests interact with cleaners.

In Section 4.2, we explore how to compute the *flake rate* for a victim test, i.e., the probability that the test fails in a randomly sampled test order of all tests in the test suite. For this example, the 392 tests could, in theory, be run in 392! ($\sim 10^{848}$) test orders (permutations), but in practice, JUnit never interleaves test methods from different test classes. These tests are split into 48 classes that actually have $\sim 10^{234}$ test orders that JUnit could run. The relevant 34 tests (1 victim, 2 polluters, and 31 cleaners) belong to 8 test classes: 2 polluters belong to one class (`TestJobHistoryEventHandler`), 11 cleaners belong to the same class as the polluters, 1 cleaner belongs to the same class as the victim (`TestMRAppMaster`), and the remaining 19 cleaners belong to six other classes. For this victim, randomly sampling the orders that JUnit could run gives a flake rate of 4.5%. In Section 4.4, we propose a simple change to increase the probability of detecting OD tests by running a reverse of each passing test order. For this victim, the conditional probability that the reverse order fails is 4.9%.

A commonly asked question is whether all detected OD tests should be fixed. While ideally all flaky tests should be fixed, some are not fixed [21,23]. For the majority of OD tests, fixing them is good to prevent flaky-test failures that can mislead the developers into debugging the wrong parts of the code; also, fixing OD tests enables tests to be run in any order, which then enables the use of beneficial regression-testing techniques [23]. Some OD tests are intentionally run in specific orders (e.g., using the `@FixMethodOrder` annotation in JUnit) to speed up testing by reusing states. We have submitted fixes for a large number of flaky tests in our prior work [19].

### 3 Preliminaries

We next formalize the concepts that we have introduced informally and define some new concepts. Let $T = \{t_1, t_2, \ldots, t_n\}$ be a set of $n$ tests partitioned in $k$ classes $\mathbb{C} = \{C_1, C_2, \ldots, C_k\}$. We use class($t$) to denote the class of test $t$. Each class $C_i$ has $n_i = |\{t \in T \mid \text{class}(t) = C_i\}|$ tests.

We use $\omega(T')$ to denote a test order, i.e., a permutation of tests in $T' \subseteq T$, and drop $T'$ when clear from the context. We use $\omega_i$ to denote the $i$-th test in the test order $\omega$, and $|\omega|$ to denote the length of a test order as measured by the number of tests. We use $t \prec_\omega t'$ to denote that test $t$ is before $t'$ in the test order $\omega$. We will analyze some cases that allow all $n!$ permutations, potentially interleaving tests from different classes. We use $\Omega_A(T)$ to denote the set of all test orders for $T$. Some testing tools [47] explore all these test orders, potentially generating false alarms because most testing frameworks [4,17,38,41] do not allow all these test orders.

We are primarily concerned with *class-compatible* test orders where all tests from each class are consecutive, i.e., if $\text{class}(\omega_i) = \text{class}(\omega_{i'})$, then for all $j$ with $i < j < i'$, $\text{class}(\omega_i) = \text{class}(\omega_j)$. We use $\Omega_C(T)$ to denote the set of all class-compatible test orders for $T$. The number of such class-compatible test orders is $k! \prod_{i=1}^{k} n_i!$. Section 4.2 presents how to compute the flake rate, i.e., the percentage of test orders in which a given victim test (with its polluters and cleaners) fails.

Section 5 presents how to systematically generate test orders to ensure that all test pairs are covered. A *test pair* $\langle t, t' \rangle$ consists of two distinct tests $t \neq t'$. We say that a test order $\omega$ *covers* a test pair $\langle t, t' \rangle$, in notation $\text{cover}(\omega, \langle t, t' \rangle)$, iff the two tests are consecutive in $\omega$, i.e., $\omega = \langle \ldots, t, t', \ldots \rangle$. Considering consecutive tests is important because a victim may not fail if not run right after a polluter, i.e., when a cleaner is run between the polluter and the victim. A set of test orders $\Omega$ covers the union of test pairs covered by each test order $\omega \in \Omega$. In general, test orders in a set can be of different lengths. Each test order $\omega$ covers $|\omega| - 1$ test pairs.

We distinguish *intra-class* test pairs, where $\text{class}(t) = \text{class}(t')$, and *inter-class* test pairs, where $\text{class}(t) \neq \text{class}(t')$. Of the total $n(n-1)$ test pairs, each class $C_i$ has $n_i(n_i - 1)$ intra-class test pairs, and the number of inter-class test pairs is $2 \sum_{1 \leq i < j \leq k} n_i n_j$. Each class-compatible test order of all $T$ tests covers $n_i - 1$ intra-class test pairs for each class $C_i$ and $k - 1$ inter-class test pairs.

We aim to generate a set of test orders $\Omega$ that cover all test pairs[3]. If we consider $\Omega_A(T)$ that allows all test orders, we need at least $n$ test orders to cover all $n(n-1)$ test pairs. When we have only one class or all classes have only one test, then all test orders are class-compatible. However, consider the more common case when we have more than one class and some class has more than one test. If we consider $\Omega_C(T)$ that allows only class-compatible test orders, we need at least $\max_{i=1}^{k} n_i$ test orders to cover all intra-class test pairs and at

[3] This problem should not be confused with *pairwise testing* [33], which typically aims to cover pairs of values from different test parameters.

least $M = 2\sum_{1\leq i<j\leq k} n_i n_j/(k-1)$ test orders to cover all inter-class test pairs; because $M > \max_{i=1}^{k} n_i$, we need at least $M$ class-compatible test orders to cover all test pairs.

More precisely, we aim to generate a set of test orders $\Omega$ that has the lowest cost for test execution. The cost for each test order $\omega$ can be modeled well as a sum of a fixed cost $\mathrm{Cost}_0$ (e.g., corresponding to the time required to start a JVM and load required classes) and a cost for each test (e.g., the time to execute the test method): $\mathrm{Cost}(\omega) = \mathrm{Cost}_0 + \sum_{t\in\omega} \mathrm{Cost}(t)$. The cost for a set of test orders is then simply the sum of individual costs $\mathrm{Cost}(\Omega) = \sum_{\omega\in\Omega} \mathrm{Cost}(\omega)$. For example, a trivial way to cover all test pairs is with a set of test orders where each test order is just a test pair: $\Omega_p = \{\langle t, t'\rangle \mid t, t' \in T \wedge t \neq t'\}$; however, the cost is unnecessarily high: $\mathrm{Cost}(\Omega_p) = n(n-1)\mathrm{Cost}_0 + 2(n-1)\mathrm{Cost}(T)$, where $\mathrm{Cost}(T) = \sum_{t\in T} \mathrm{Cost}(t)$.

To simplify, we can assume that each test in $T$ has the same cost, say, $\mathrm{Cost}_1$, and then $\mathrm{Cost}(\Omega_p) = n(n-1)\mathrm{Cost}_0 + 2n(n-1)\mathrm{Cost}_1$. In the optimal case, each test order would be a permutation of $n$ tests covering $n-1$ test pairs, and the number of test orders would be just $n(n-1)/(n-1) = n$. Therefore, the lowest cost is $\mathrm{Cost}(\Omega_{opt}) = n\mathrm{Cost}_0 + n^2\mathrm{Cost}_1$, demonstrating that the factor for $\mathrm{Cost}_0$ can be substantially reduced, while the factor for $\mathrm{Cost}_1$ is nearly halved $(\frac{n}{2(n-1)})$. However, in most realistic cases, due to the constraints of class-compatible test orders and the big differences in the number of tests across different classes, we cannot reach the optimal case.

#### 3.1 Dataset for Evaluation

Besides deriving some analytical results, we also run some empirical experiments on flaky tests from Java projects. Our recent work [25] ran the iDFlakies tool on most test suites in the projects from the iDFlakies dataset [15] using the configurations recommended by our iDFlakies work [22]. Specifically, we ran 100 randomly sampled test orders from $\Omega_C(T)$ and 1 test order that is the reverse order of what Maven Surefire [29] runs by default. Note that unlike our work in Section 4.4, where we propose running a reverse test order of *every* test order where all tests passed, the one reverse order that we ran in our recent work [25] may or may not have been from a passing test order, and the reverse order is run only once and not for every passing test order.

Each project in the iDFlakies dataset is a Maven-based, Java project organized into one or more *modules*, which are (sub)directories that organize code under test and test code. Each module contains its own test suite. For the remainder of the paper, we use the 121 modules in which our recent work [25] found at least one flaky test (but not necessarily OD test). To illustrate diversity among these 121 modules, the number of classes ranges from 1 to 2215, with an average of 61, and the total number of tests ranges from 1 to 4781, with an average of 287. The number of tests per class ranges from 1 to 200, with an average of 4.8.

When we run some of the test orders generated by our systematic test-pair exploration as described in Section 5.2, we detect a total of 249 OD tests in 44

of the 121 modules. Of the 249 OD tests, 57 are brittles and 192 are victims. Compared to the OD tests detected in our prior work [22,24,25] that used the iDFlakies dataset, we find 44 new OD tests that have not been detected before. Of the 44 OD tests, 1 is brittle and 43 are victims. One of the newly detected victim tests (`testMRAppMasterSuccessLock`) is shown in Section 2.

## 4 Analysis of Flake Rate and Simple Algorithm Change

We next discuss how to compute the flake rate for each OD test. Let $T$ be a test suite with an OD test. Prior work [22,24,25,47] would run many test orders of $T$ and compute the flake rate for each test as a ratio of the number of test failures and the number of test runs. However, failures of flaky tests are probabilistic, and running even many test orders may not suffice to obtain the true flake rate for each test. Running more test orders is rather costly in machine time; in the limit, we may need to run all $|T|!$ permutations to obtain the true flake rate for OD tests. To reduce machine time needed for computing the flake rate for OD tests, we first propose a new procedure, and then derive formulas based on this procedure. We finally show a simple change for sampling random test orders to increase the probability of detecting OD tests.

### 4.1 Determining Test Outcome without Running a Test Order

We use a two-step procedure to determine the test outcome for a given OD test. We assume that some prior runs already detected the OD test, and the goal is to determine the test outcome for some new test orders that were not run.

In Step 1, we classify how each test from $T$ relates to each OD test in a simple setting that runs only *up to three* tests. Specifically, we first determine whether an OD test $t$ is a victim or a brittle by running the test in isolation, i.e., just $\langle t \rangle$, by itself 10 times: if $t$ always passes, it is considered a victim (although it may be an ND test); if $t$ always fails, it is considered a brittle (although it may be an ND test); and if $t$ sometimes passes and sometimes fails, it is definitely an ND, not OD, test. This approach was proposed for iFixFlakies [37], and using 10 runs is a common industry practice to check whether a test is flaky [31,40].

We then find (1) for each victim, *all* its single polluters in $T$ and also *all* single cleaners for each polluter, and (2) for each brittle, *all* its single state-setters in $T$. To find polluters (resp. state-setters) of a victim (resp. brittle) test, iFixFlakies [37] takes as input a test order (of entire $T$) where the test failed (resp. passed) and then searches the prefix of the test in that test order using delta debugging [46] (an extended version of binary search). While iFixFlakies can find all polluters (resp. state-setters) in the prefix, it does not necessarily find all polluters in $T$, and it takes substantial time to find these polluters using delta debugging. The experiments show that in 98% of cases, binary search finds one test to be a polluter, although some rare cases need a *polluter group* that consists of two tests.

We propose a simpler and faster approach to find polluters (resp. state-setters) for the most common case: for each victim $v$ (resp. brittle $b$) and each test $t \in T \setminus \{v\}$ (resp. $t \in T \setminus \{b\}$), we run a pair of the test and the victim (resp. brittle), i.e., $\langle t, v \rangle$ (resp. $\langle t, b \rangle$). If the victim fails (resp. brittle passes), then the test $t$ is a polluter (resp. state-setter). Further, for each victim $v$, its polluter $p$, and a test $t \in T \setminus \{v, p\}$, we run a triple of $\langle p, t, v \rangle$, and if $v$ passes, then $t$ is a cleaner for the pair of $v$ and $p$. Note that for the same victim $v$, different polluters may have different cleaners such as the example presented in Section 2.

In Step 2, we determine whether each OD test passes or fails in a given test order using only the abstraction from Step 1, without actually running the test order. We focus on victims because they are more complex than brittles; brittles can be viewed as special cases with slight changes (requiring a state-setter to *run* before a brittle to pass, rather than requiring a polluter *not* to run before a victim to pass). Without loss of generality, we consider one victim at a time. Intuitively, the victim fails in a test order if a polluter is run before the victim without a cleaner between the polluter and the victim. Formally, we define the test outcome as follows.

**Definition 1 (Test Outcome from Abstraction).** *Let $T$ be a test suite with one victim $v \in T$, polluters $P \subset T$, and a family of cleaners $C_p \subset T$ indexed by each polluter $p \in P$. The outcome of $v$ in a test order $\omega$ is defined as follows:*

$$\text{fail}(\omega) \equiv \exists p \in P.\, p \prec_\omega v \wedge \nexists c \in C_p.\, p \prec_\omega c \wedge c \prec_\omega v; \quad \text{pass}(\omega) \equiv \neg\text{fail}(\omega).$$

This definition is an estimate of what one would obtain for all (repeated) runs of $|T|!$ permutations, for three main reasons: (1) tests may behave differently in test orders than in isolation [24] (and an OD test may even be an ND test in some orders [24]); (2) polluters, cleaners, and state-setters may not be single tests but groups (iFixFlakies [37] reports that groups are rather rare); and (3) a test that fails in some prefix may behave differently for the tests that come after it in a test order than when the test passes (again, iFixFlakies [37] reports this issue to be rare, finding just one such case). Despite these potential sources of error, our evaluation shows that our use of abstraction obtains flake rates similar to iDFlakies for orders that iDFlakies ran. Most importantly, our use of abstraction allows us to evaluate many more orders without actually running them, thus taking much less machine time.

#### 4.2 Computing Flake Rate

We next define flake rate, derive formulas for computing flake rate for two cases, and show why we need to sample test orders for other cases.

**Definition 2 (Flake Rate).** *For a test suite $T$ with exactly one victim, given a set of test orders $\Omega(T)$, the flake rate is defined as the ratio:*

$$f(T) = |\{\omega \in \Omega(T) \mid \text{fail}(\omega)\}| \,/\, |\Omega(T)|;$$

*we use the subscript $f_A$ and $f_C$ when we need to refer specifically to the flake rate for $\Omega_A(T)$ and $\Omega_C(T)$ (defined in Section 3), respectively.*

We derive the formula for flake rate based on the number of polluters $P$ and cleaners $C$ for two special cases. In general, computing the flake rate can ignore tests that are not *relevant*, i.e., not in $\{v\} \cup P \cup \bigcup_{p \in P} C_p$. It is easy to prove that $f(T) = f(T')$ if $T$ and $T'$ have the same victim, polluters, and cleaners—the reason is that the tests from $T \setminus T'$ are irrelevant in any order and do not affect the outcome of $v$; we omit the proof due to space limit. The further analysis thus focuses only on the relevant tests.

**Special Case 1:** Assume that (A1) all polluters have the same set $C$ of cleaners: $C = C_p, \forall p \in P$; and (A2) all of the victim, polluters, and cleaners are in the same class: $\forall t, t' \in \{v\} \cup P \cup C.\text{class}(t) = \text{class}(t')$; it means that $\Omega_A(T) = \Omega_C(T)$ and $f_A = f_C$. Let $\pi = |P|$ and $\gamma = |C|$. The total number of permutations of the relevant tests is $(\pi + \gamma + 1)!$. While we can obtain $|\{\omega \in \Omega(T) \mid \text{fail}(\omega)\}|$ purely by definition, counting test orders where the victim fails, we prefer to take a probabilistic approach that will simplify further proofs. A victim fails if (1) it is not in the first position, with probability $(\pi + \gamma)/(\pi + \gamma + 1)$, and (2) its immediate predecessor is a polluter, with probability $\pi/(\pi + \gamma)$, giving the overall flake rate $f(T) = \pi/(\pi + \gamma + 1)$. This formula is simple, but real test suites often violate A1 or A2. Of the 249 tests used in our experiments, 13 violate both A1 and A2, 207 violate only A2, and only 29 do not violate either.

**Special Case 2:** Keeping A1 but relaxing A2, assume that the victim is in class $C_1$ with $\pi_1$ polluters and $\gamma_1$ cleaners, and the other $k-1$ classes have $\pi_i$ polluters and $\gamma_i$ cleaners, $2 \leq i \leq k$, where in general, either $\pi_i$ or $\gamma_i$, but not both, can be zero for any class except for the victim's own class where both $\pi_1$ and $\gamma_1$ can be zero. Per Special Case 1, we have $f_A(T) = (\sum_{i=1}^{k} \pi_i)/(\sum_{i=1}^{k} \pi_i + \sum_{i=1}^{k} \gamma_i + 1)$. Next, consider class-compatible test orders, which do not interleave tests from different classes. The victim fails if (1) it fails in its own class, with probability $\pi_1/(\pi_1 + \gamma_1 + 1)$, or (2) the following three conditions hold: (2.1) the victim is the first in its own class, with probability $1/(\pi_1 + \gamma_1 + 1)$, (2.2) the class is *not* the first among classes, with probability $(k - 1)/k$, and (2.3) the immediately preceding class ends with a polluter, with probability $\pi_i/(\pi_i + \gamma_i)$ for each class $i$ and thus the probability $\sum_{i=2}^{k}(\pi_i/(\pi_i + \gamma_i))/(k - 1)$ across all classes. Overall,

$$f_C(T) = \frac{\pi_1 + \frac{1}{k}\sum_{i=2}^{k}\frac{\pi_i}{\pi_i + \gamma_i}}{\pi_1 + \gamma_1 + 1}.$$

The formula is already more complex. It is important to note that we can have either $f_A(T) \geq f_C(T)$ or $f_C(T) \geq f_A(T)$, based on the ratio of polluters and cleaners in the victim's own class vs. the ratio of polluters and victims in other classes, i.e., neither set of test orders ensures a higher flake rate. We show in Section 4.3 that both cases arise in practice.

**General Case:** In the most general case, relaxing A1 to allow different polluters to have a different set of cleaners, while also having all these relevant tests in different classes, it appears challenging to derive a closed-form expression for $f_A(T)$, let alone for $f_C(T)$. We thus resort to estimating flake rates by sampling orders from $\Omega_A(T)$ or $\Omega_C(T)$, and counting what ratio of them fail based on Definition 1 in Section 4.3.

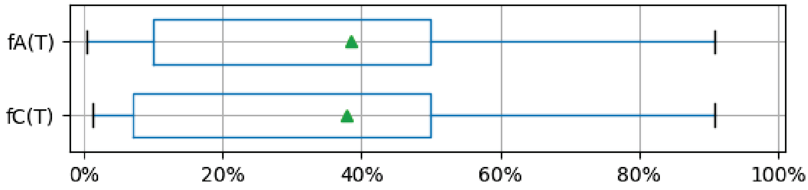

**Fig. 3.** Distribution of flake rate for two sets of test orders.

#### 4.3 Comparing Flake Rate for Different Sets of Test Orders

While tools such as iDFlakies [22] incorporate the requirement of not interleaving tests from different classes in a test order, some other tools [47] do not incorporate this requirement, so they allow all test orders. Recall that $\Omega_A(T)$ denotes the set of all test orders and $\Omega_C(T)$ denotes the set of test orders that satisfy the requirement. The reason to run $\Omega_A(T)$ is to try to maximize the detection of all potential OD tests at the risk that some detected failures would be false positives. In particular, a test failure observed in some non-class-compatible order may not be reproducible in any class-compatible prefix of that order, e.g., due to the various ways to customize JUnit [17] (with annotations such as `@Before`, `@BeforeClass`, `@Rule`) or similar testing frameworks. The reason to run only $\Omega_C(T)$ is to detect OD-test failures that developers can observe from running the tests and are therefore motivated to fix.

While both sets of test orders can detect all true positive OD tests, it is not clear which set of test orders are *more likely* to detect *true positive* OD tests. Intuitively, running $\Omega_A(T)$ test orders can more likely detect failures if cleaners and victims are in the same class, while polluters are in different classes; in such cases, polluters are less likely to come in between cleaners and the victim. For example, for the victim presented in Section 2, the $\Omega_A(T)$ flake rate is 10.5%, while the $\Omega_C(T)$ flake rate is 4.5%. On the other hand, running $\Omega_C(T)$ test orders can more likely detect failures if polluters and victims are in the same class, while cleaners are in different classes. Similar reasoning applies to brittles: if state-setters are more often in the same test class as the brittle, then the brittle is less likely to fail than if state-setters are more often in other classes.

To compare these sets of test orders on real OD tests, we use the dataset of 192 victim and 57 brittle tests described in Section 3.1. We collect all single test polluters for each victim and all single test cleaners for each polluter-victim pair. We also collect all single test state-setters for the brittles. We then use either the formulas presented in Section 4.2 or a large number of uniformly sampled test orders to obtain the flake rates, $f_A(T)$ and $f_C(T)$, for each test. Specifically, our formulas apply for 236 of the 249 tests. For the remaining 13 tests (all victims), we sample 100,000 test orders from each of $\Omega_A(T)$ and $\Omega_C(T)$ to estimate their flake rates.

Figure 3 summarizes the results. For each set of test orders, the figure shows a boxplot that visualizes the distribution of flake rates for 249 OD tests. The

$f_A(T)$ flake rates have a slightly higher mean (38.4%) than the $f_C(T)$ flake rates (38.0%). Statistical tests for paired samples of the flake rates—specifically, dependent Student's t-test obtains a $p$-value of 0.47 and Wilcoxon signed-rank test obtains a $p$-value of 0.01—show that the differences could be statistically significant (at $\alpha = 0.05$ level). However, if we omit the 13 tests that required samplings, the means are 38.3% for $f_A(T)$ and 38.6% for $f_C(T)$, and the difference is not statistically significant (dependent Student's t-test obtains a $p$-value of 0.55, and Wilcoxon signed-rank test obtains a $p$-value of 0.19).

Prior work [6,22,24,47] has not performed any explicit comparison between the two sets of test orders. Our results demonstrate that running $\Omega_A(T)$ might be more likely to detect true positive OD tests. However, using such test orders may contain false positives. Future work on detecting OD tests should explore how to address false positives if $\Omega_A(T)$ test orders are run.

#### 4.4 Simple Change to Increase Probability of Detecting OD Tests

Inspired by our probability analysis, we propose a simple change to increase the probability of detecting OD tests. The standard algorithm for sampling $S$ random test orders simply repeats $S$ times the following steps: (1) $\omega \leftarrow$ sample a random test order from possible test orders ($\Omega_A(T)$ or $\Omega_C(T)$); (2) obtain result $r \leftarrow \text{run}(\omega)$; (3) if $r$ is FAIL, then print $\omega$. (A variant [22] may store previously sampled test orders to avoid repetition, but the number of possible test orders is usually so large that sampling the same one is highly unlikely, so one can save space and time by not tracking previously sampled test orders.)

Our key change is to select the next test order as a *reverse* of the prior test order that passed: (4) if $r$ is PASS, then $\omega_R \leftarrow \text{reverse}(\omega)$. The intuition for this change is that a passing order may have the polluter *after* the victim. Therefore, reversing the passing order would have the polluter *before* the victim, and thus the reverse of the passing order should have a higher probability to fail than a random order that may have the polluter before or after the victim. Note that the reverse of a class-compatible test order is also a class-compatible test order, so this change applies to $\Omega_C(T)$. The other changes are to run $\omega_R$, print if it fails, and properly count the test orders to select exactly $S$ samples of test orders.

We next compute the probability that the reverse of a passing order fails. **Special Case 1:** Consider the Special Case 1 scenario from Section 4.2 with $\pi$ polluters and $\gamma$ cleaners. For the standard algorithm, $f(T) = f_A(T) = f_C(T) = \pi/(\pi + \gamma + 1)$. For our change, the conditional probability that the second test order fails given that the first test order passes is $P(\text{fail}(\omega_R)|\text{pass}(\omega)) = P(\text{fail}(\omega_R) \wedge \text{pass}(\omega))/P(\text{pass}(\omega))$. We already have $P(\text{pass}(\omega)) = 1 - f(T) = (\gamma + 1)/(\pi + \gamma + 1)$.

To compute $P(\text{fail}(\omega_R) \wedge \text{pass}(\omega))$, we consider two cases based on the position of the victim in the passing test order $\omega$. (1) If the victim is first, with the probability of $1/(\pi + \gamma + 1)$, then the second test should be a polluter, with the probability of $\pi/(\pi + \gamma)$, so we get $\pi/((\pi + \gamma)(\pi + \gamma + 1))$ for this case. (2) If the victim is not first, it cannot be the last in $\omega$ because otherwise, $\omega_R$ would not fail, so the victim is in the middle, with the probability of $(\pi + \gamma - 1)/(\pi + \gamma + 1)$.

We also need a cleaner right before the victim, with probability $\gamma/(\pi+\gamma)$, and a polluter right after the victim, with probability $\pi/(\pi+\gamma-1)$. Overall, we get the probability $\pi\gamma/((\pi+\gamma)(\pi+\gamma+1))$ for this case. We can sum up the two cases to get $P(\text{fail}(\omega_R) \wedge \text{pass}(\omega)) = \pi(\gamma+1)/((\pi+\gamma)(\pi+\gamma+1))$.

Finally, the conditional probability that the reverse test order fails given the first test order passes is $P(\text{fail}(\omega_R)|\text{pass}(\omega)) = (\frac{\pi(\gamma+1)}{(\pi+\gamma)(\pi+\gamma+1)})/(\frac{\gamma+1}{\pi+\gamma+1}) = \pi/(\pi+\gamma)$. This probability is strictly larger than $f(T) = \pi/(\pi+\gamma+1)$, because $\pi > 0$ must be true for the victim to be a victim.

**Special Case 2:** For the Special Case 2 scenario from Section 4.2, the common case is $\pi_1 + \gamma_1 > 0$ (i.e., the victim's class $C_1$ has at least one other relevant test). Based on the relative position of the victim in class $C_1$, we consider three cases: the victim runs first, in the middle, or last in class $C_1$. After calculating the probability for the three cases separately and summing them up, we get the probability that the reverse test order fails and the first test order passes as $P(\text{fail}(\omega_R) \wedge \text{pass}(\omega)) = \frac{\pi_1+k\pi_1\gamma_1+\pi_1 S_\gamma+\gamma_1(\pi_1+\gamma_1+1)S_\pi}{k(\pi_1+\gamma_1)(\pi_1+\gamma_1+1)}$ where $S_\pi = \sum_{i=2}^{k} \frac{\pi_i}{\pi_i+\gamma_i}$ and $S_\gamma = \sum_{i=2}^{k} \frac{\gamma_i}{\pi_i+\gamma_i}$. In Section 4.2, we have computed $P(\text{pass}(\omega))$, so dividing $P(\text{fail}(\omega_R) \wedge \text{pass}(\omega))$ by $P(\text{pass}(\omega))$ gives the conditional probability that the reverse test order fails given the first test order passes. Due to the complexity of the formulas, it is difficult to show a detailed proof that $P(\text{fail}(\omega_R)|\text{pass}(\omega)) > f(T)$, so we sample test orders instead.

When we sample both $\Omega_A(T)$ and $\Omega_C(T)$ for 100,000 random test orders on all 249 OD tests without reverse (i.e., the standard algorithm) and with reverse when a test order passes (i.e., our change), we find that our change does statistically significantly increase the chance to detect OD tests. Specifically, for $\Omega_A(T)$, test orders without reverse obtain a mean of 38.6%, while test orders with reverse of passing test orders obtain a mean of 45.3%. Statistical tests for paired samples on the flake rates without and with reverse for $\Omega_A(T)$ show a $p$-value of $\sim 10^{-38}$ for dependent Student's t-test and a $p$-value of $\sim 10^{-43}$ for Wilcoxon signed-rank test. Similarly, for $\Omega_C(T)$, test orders without reverse obtain a mean of 38.0%, while test orders with reverse of passing test orders obtain a mean of 45.3%. Statistical tests for paired samples on the flake rates without and with reverse for $\Omega_C(T)$ show a $p$-value of $\sim 10^{-42}$ for dependent Student's t-test and a $p$-value of $\sim 10^{-42}$ for Wilcoxon signed-rank test.

Based on these positive results, we have changed the iDFlakies tool [22] so that, by default, it runs the reverse of the previous order, instead of running a random order, if the previous order found no new flaky test.

### 5 Generating Test Orders to Cover Test Pairs

We next discuss our algorithm to generate test orders that systematically cover all test pairs for a given set $T$ with $n$ tests. The motivation is that even with our change to increase the probability to detect OD tests, the randomization-based sampling remains inherently probabilistic and can fail to detect an OD test.

#### 5.1 Special Case: All Orders are Class-Compatible

We first focus on the special case where we have only one class, or many classes that each have only one test, so all $n!$ permutations are class-compatible. For example, for $n = 2$ we can cover both pairs with $\Omega_2 = \{\langle t_1, t_2\rangle, \langle t_2, t_1\rangle\}$, and for $n = 4$ we can cover all 12 pairs with 4 test orders $\Omega_4 = \{\langle t_1, t_4, t_2, t_3\rangle, \langle t_2, t_1, t_3, t_4\rangle, \langle t_3, t_2, t_4, t_1\rangle, \langle t_4, t_3, t_1, t_2\rangle\}$. Recall that $n$ is the minimum number of test orders needed to cover all test pairs, so the cases for $n = 2$ and $n = 4$ are optimal. The reader is invited to consider for $n = 3$ whether we can cover all 6 test pairs with just 3 test orders. The answer is upcoming in this section.

To address this problem, we consider *Tuscan squares* [7], objects studied in the field of combinatorics. Given a natural number $n$, a Tuscan square consists of $n$ rows, each of which is a permutation of the numbers $\{1, 2, \ldots, n\}$, and every pair $\langle i, j\rangle$ of distinct numbers occurs consecutively in some row. Tuscan squares are sometimes called "row-complete Latin squares" [34], but note that Tuscan squares need *not* have each column be a permutation of all numbers.

A Tuscan square of size $n$ is equivalent to a decomposition of the complete graph on $n$ vertices, $K_n$, into $n$ Hamiltonian paths [42]. The decomposition for even $n$ has been known since the $19^{\text{th}}$ century and is often attributed to Walecki [26]. The decomposition for odd $n \geq 7$ was published in 1980 by Tillson [42]. Tillson presented a beautiful construction for $n = 4m + 3$ and a rather involved construction for $n = 4m + 1$ with a recursive step and manually constructed base case for $n = 9$. In brief, Tuscan squares can be constructed for all values of $n$ except $n = 3$ or $n = 5$. We did not find a public implementation for generating Tuscan squares, and considering the complexity of the case $n = 4m+1$ in Tillson's construction, we have made our implementation public [44].

We can directly translate permutations from Tuscan squares into $n$ test orders that cover all test pairs in this special case (where all test pairs are either only intra-class test pairs of one class or only inter-class test pairs of $n$ classes). These sets of test orders have the minimal possible cost: $\text{Cost}(\Omega_n) = n(\text{Cost}_0 + \text{Cost}(T))$, substantially lower than $\text{Cost}(\Omega_p)$ for running all test pairs in isolation. For $n = 3$ and $n = 5$, we have to use 4 and 6 test orders, respectively, to cover all test pairs. For example, for $n = 3$ we can cover all 6 pairs with 4 orders $\{\langle t_1, t_2, t_3\rangle, \langle t_2, t_1, t_3\rangle, \langle t_3, t_1\rangle, \langle t_3, t_2\rangle\}$.

#### 5.2 General Case

Algorithm 1 shows the pseudo-code algorithm to generate test orders that cover all test pairs in the general case where we have more than one class and at least one class has more than one test. The main function calls two functions to generate test orders that cover intra-class and inter-class test pairs.

The function `cover_intra_class_pairs` generates test orders that cover all intra-class test pairs. For each class, the function `compute_tuscan_square` is used to generate test orders of tests within the class to cover all intra-class test pairs. These test orders for each class are then appended to form a test order for the entire test suite $T$. The function `pick`, invoked on multiple lines,

**Algorithm 1:** Generate test orders that cover all intra-test-class and inter-test-class test-method pairs

```
Input: T          # test suite, a set of test methods partitioned into test classes
Output: Ω                                           # output is a set of test orders
Function cover_all_pairs():
    Ω = {}                                                              # empty set
    cover_intra_class_pairs()
    cover_inter_class_pairs()
Function cover_intra_class_pairs():
    map = {}                             # map each class to all its intra-class orders
    for C ∈ classes(T) do
        map = map ∪ {⟨C, ω_C⟩ | ω_C ∈ compute_tuscan_square(C)}
    while map ≠ {} do
        ω = ⟨⟩                                                          # empty order
        Cs = {C | ∃ω_C. ⟨C, ω_C⟩ ∈ map}
        for C ∈ Cs do
            ω_C = pick({ω_C | ⟨C, ω_C⟩ ∈ map})
            map = map \ {⟨C, ω_C⟩}
            ω = ω ⊕ ω_C                                                 # append order
        Ω = Ω ∪ {ω}
Function cover_inter_class_pairs():
    pairs = {⟨t, t′⟩ | t, t′ ∈ T ∧ class(t) ≠ class(t′)}\ # from all inter-class pairs..
       {⟨t, t′⟩ | ∃ω ∈ Ω. cover(ω, ⟨t, t′⟩)} # ..remove covered by intra-class orders
    while pairs ≠ {} do
        ω = pick(pairs)          # start with a randomly chosen not-covered pair
        pairs = pairs \ {ω}
        while true do
            t_p = ω_{|ω|−1}                                     # previously last test
            ts = {t | ⟨t_p, t⟩ ∈ pairs ∧ class(t) ∉ classes(ω)}
            if ts = {} then
                break
            t_n = pick(ts)                                  # next test to extend order
            pairs = pairs\{⟨t_p, t_n⟩}
            ω = ω ⊕ t_n
        Ω = Ω ∪ {ω}
```

chooses a random element from a set. The outer loop iterates as many times as the maximum number of intra-class test orders for any class. When the loop finishes, $\Omega$ contains a set of test orders that cover *all intra-class* and *some inter-class* test pairs. Each test order that concatenates tests from $l$ classes covers $l-1$ inter-class test pairs. (Using just these test orders, we already detected 44 new OD tests in the test suites from the iDFlakies dataset.) Each intra-class test pair is covered by exactly one test order. Modulo the special cases for $n = 3$ and $n = 5$, each *covered* inter-class pair appears in *exactly one* test order in $\Omega$, because Tuscan squares satisfy the invariant that each element appears only once as the first and once as the last in the permutations in a Tuscan square.

The function `cover_inter_class_pairs` generates more test orders to cover the remaining inter-class test pairs. It uses a greedy algorithm to first initialize a test order with a randomly selected not-covered test pair and then extend the test order with a randomly selected not-covered test pair as long as an appropriate test pair exists. Extending the test order as long as possible reduces both the number of test orders and the number of times each test needs to be run.

We evaluate our randomized algorithm on 121 modules from the iDFlakies dataset as described in Section 3.1. We use the total cost, which considers the number of test orders and the number of tests in all of those test orders. The number of test orders is related to $\text{Cost}_0$, while the number of tests is related to $\text{Cost}_1$ as defined in Section 3. We run our algorithm 10 times for various random seeds. The coefficient of variation [3] for each module shows that the algorithm is fairly stable, with the average for all modules being only 1.1% and 0.25% for the number of test orders and the number of tests, respectively.

Compared with $\Omega_p$ that has all test orders of just test pairs, our randomized algorithm's average number of test orders and the average number of tests are only 3.68% and 51.8%, respectively, that of all the $\Omega_p$ test orders. The overall cost of the test orders generated by our randomized algorithm is close to the optimal, because the number of test orders is reduced by almost two orders of magnitude, and 51.8% of the number of tests is close to the theoretical minimum of 50% that of $\Omega_p$ test orders for $\text{Cost}_1$.

### 6 Conclusion

Order-dependent (OD) tests are one prominent category of flaky tests. Prior work [22,24,47] has used randomized test orders to detect OD tests. In this paper, we have presented the first analysis of the probability that randomized test orders detect OD tests. We have also proposed a simple change for sampling random test orders to increase the probability of detecting OD tests. We have finally proposed a novel algorithm that systematically explores all consecutive pairs of tests, guaranteeing to find all OD tests that depend on one other test. Our experimental results show that our algorithm runs substantially fewer tests than a naive exploration that runs all pairs of tests. Our runs of some test orders generated by the algorithm detect 44 new OD tests, not detected in prior work [22,24,25] on the same evaluation dataset.

### Acknowledgments

We are grateful to Peter Taylor for a StackExchange post [39] that led us to the concept of Tuscan squares. We thank Dragan Stevanović, Wenyu Wang, and Zhengkai Wu for discussions about Tuscan squares and Reed Oei for comments on the paper draft. This work was partially supported by NSF grants CNS-1564274, CNS-1646305, CCF-1763788, and CCF-1816615. We also acknowledge support for research on flaky tests from Facebook and Google.
